# Impact of brain overgrowth on sensorial learning processing during the first year of life

**DOI:** 10.3389/fnhum.2022.928543

**Published:** 2022-07-19

**Authors:** Gabriela López-Arango, Florence Deguire, Kristian Agbogba, Marc-Antoine Boucher, Inga S. Knoth, Ramy El-Jalbout, Valérie Côté, Amélie Damphousse, Samuel Kadoury, Sarah Lippé

**Affiliations:** ^1^Research Center, Sainte-Justine Hospital, Montreal University, Montreal, QC, Canada; ^2^Department of Neurosciences, Montreal University, Montreal, QC, Canada; ^3^Department of Psychology, Montreal University, Montreal, QC, Canada; ^4^Polytechnique Montreal, Montreal, QC, Canada; ^5^Department of Medical Imaging, Sainte-Justine Hospital, Montreal University, Montreal, QC, Canada

**Keywords:** repetition suppression, change detection, macrocephaly, time-frequency analysis, ERPs (event related potentials)

## Abstract

Macrocephaly is present in about 2–5% of the general population. It can be found as an isolated benign trait or as part of a syndromic condition. Brain overgrowth has been associated with neurodevelopmental disorders such as autism during the first year of life, however, evidence remains inconclusive. Furthermore, most of the studies have involved pathological or high-risk populations, but little is known about the effects of brain overgrowth on neurodevelopment in otherwise neurotypical infants. We investigated the impact of brain overgrowth on basic perceptual learning processes (repetition effects and change detection response) during the first year of life. We recorded high density electroencephalograms (EEG) in 116 full-term healthy infants aged between 3 and 11 months, 35 macrocephalic (14 girls) and 81 normocephalic (39 girls) classified according to the WHO head circumference norms. We used an adapted oddball paradigm, time-frequency analyses, and auditory event-related brain potentials (ERPs) to investigate differences between groups. We show that brain overgrowth has a significant impact on repetition effects and change detection response in the 10–20 Hz frequency band, and in N450 latency, suggesting that these correlates of sensorial learning processes are sensitive to brain overgrowth during the first year of life.

## Introduction

Macrocephaly, defined as an occipitofrontal head circumference exceeding the 97th percentile, is considered a relatively common condition found in about 2–5% of the infant population (Tan et al., 2018; Jones and Samanta, 2020). A benign form is observed when macrocephaly is an isolated and familial trait, however, in some cases it precedes a genetic, congenital, or acquired pathological condition (Tan et al., 2018; Jones and Samanta, 2020). Some studies have suggested that an increased brain parenchyma or brain overgrowth is associated with neurodevelopmental disorders such as autism (Courchesne et al., 2003, 2005; Courchesne and Pierce, 2005; Bigler et al., 2010; Rommelse et al., 2011; Dougherty et al., 2016; Piven et al., 2017). Indeed, an increased brain overgrowth rate has been reported in population with autism (20 vs. 3% in neurotypical population), specially during the first year of life (Lainhart et al., 1997, 2006).

A recent study on the relationship between brain overgrowth and neurodevelopmental disorders such as autism, also emphasizes the presence of alterations in basic attentional and sensorimotor processing at the end of the first year of life (Piven et al., 2017). The strength of this association and the mechanisms involved are still under investigation.

Hence, most studies have investigated macrocephaly in the context of clinical conditions. To our knowledge only one study has investigated the impact of macrocephaly on the neurodevelopment of the general population, showing a significant association between atypical head circumference growth during the first year of life and behavioral traits at 24 months (Dupont et al., 2018). In this study, the authors found that greater head circumference growth predicts lower temperamental effortful control and lower surgency/extraversion in boys (Dupont et al., 2018). These intriguing findings suggest that greater head circumference growth needs further investigation not only in clinical populations but also in the neurotypical population.

Brain growth during the first year of life is critical and involves cue developmental mechanisms such as migration, synaptogenesis, gliogenesis, myelination, apoptosis, and circuits formation that underlie perceptual, cognitive, and behavioral skills in infants (Chugani, 1998; van Dyck and Morrow, 2017).

During infancy, perceptual and cognitive development have been studied through habituation and novelty paradigms (Bornstein and Benasich, 1986; Colombo and Mitchell, 2009; Oakes, 2010; Kavsek, 2013). Habituation and novelty response constitute the simplest form of learning also called sensory learning and some studies have shown that these responses are sensitive to neuronal alterations (Colombo and Mitchell, 2009; Kavsek, 2013). The repetition suppression effect (decrease in neural response) (Turk-Browne et al., 2008) and the change detection response (increase in neural response) (mismatch negativity) (Naatanen and Alho, 1997) are electrophysiological brain responses of sensory learning observed already during the first year of life (Dehaene-Lambertz and Dehaene, 1994; Cheour et al., 2000; Snyder and Keil, 2008; Basirat et al., 2014). However, not only a repetition suppression but also a repetition enhancement response has been reported across the life span (Karhu et al., 1997; Bouchon et al., 2015; Recasens et al., 2015). Repetition enhancement has been proposed as a complementary process of repetition suppression also involved in memory trace consolidation (Karhu et al., 1997; Recasens et al., 2015). We will refer to repetition suppression and repetition enhancement as repetition effects.

Repetition effects and change detection response have provided significant insights into different perceptual and cognitive processes in infants, including perceptual discrimination, memory, attention, and language, among others (Snyder and Keil, 2008; He et al., 2009b; Háden et al., 2016; Nordt et al., 2016). For that reason, their potential value as early markers of neurodevelopment have been emphasized (Naatanen et al., 2014; Nordt et al., 2016). Whether these sensory learning responses, fundamental to more complex cognitive processing, are affected by brain overgrowth needs to be investigated.

The purpose of our study was to investigate whether repetition effects and change detection response differ between normocephalic and macrocephalic infants during the first year of life. Infants were classified based on their head circumference measure and the World Health Organisation norms (WHO norms). We used an oddball paradigm, which consist in presenting a frequent stimulus termed “standard,” interspersed with a rare different stimulus referred to as “deviant” or “oddball” (Polich, 2004; Kushnerenko et al., 2007). Given that the AAAX design have been successfully used in infants to study repetition effects and change detection response (Dehaene-Lambertz and Dehaene, 1994; Basirat et al., 2014; Mahmoudzadeh et al., 2017), we used an adapted paradigm from Basirat and colleagues (Basirat et al., 2014) that involves the learning of a standard sequence of vowels (/a/a/a/i/). An audio-visual presentation was chosen with the aim to maximize attention and equalize attentional involvement among participants; however, we focused our analysis on the auditory response.

Repetition effects and change detection response were investigated through event-related potentials (ERPs) and brain oscillations given that these methods may provide complementary information about the underlying mechanisms of these responses. ERPs components, obtained by averaging time-locked electrocortical responses to the onset of a stimulus, are thought to reflect the summation of neural activity of multiple generators (Shah et al., 2004; Musacchia et al., 2015). The oscillatory brain analysis involves the decomposition of evoked activity in EEG frequencies ranging from delta to gamma. The phase and the power information can be distinguished. Phase information represent the phase synchronization of EEG frequency oscillations across trials. In a complementary manner, analysis of power changes includes not only phase-locked but also non-phased-locked or induced activity. Non-phase locked activity is relevant to event related responses since it is induced by the stimulus, although eliminated in a traditional ERPs analysis by the signal averaging procedure involved in that method (Musacchia et al., 2015). Most robust response in terms of brain oscillations associated with repetition and change detection has been reported in the theta oscillations (Isler et al., 2012; Musacchia et al., 2015; Thébault-Dagher et al., 2020; López-Arango et al., 2021); however, decreases in power associated with repetition and increases in power associated with change detection in beta, alpha and gamma oscillations have also been described in neurotypical newborns (Isler et al., 2012) and in infants (Ortiz-Mantilla et al., 2013, 2016) and in preterm neonates (Mahmoudzadeh et al., 2017).

With respect to ERPs, the complex P150-N250-P350-N450 has been reported as the predominant auditory pattern of response during the first year of life (Novak et al., 1989; Kushnerenko et al., 2002; Wunderlich et al., 2006). The study of these components and their sensitivity to repetition and change detection has allowed the exploration of maturation and functional integrity of the auditory cortex in infants, critical for future development of speech and language. Indeed, auditory ERP responses to repetition and change detection have shown to be influenced by age and by risk factors such as familial risk for autism or language learning impairment (Dehaene-Lambertz and Dehaene, 1994; Cheour et al., 2000; Guiraud et al., 2011; Cantiani et al., 2016; Chen et al., 2016; Kolesnik et al., 2019).

Given that alterations in repetition suppression and change detection response have been reported in several pathological and neurodevelopmental conditions (Baldeweg, 2006; Guiraud et al., 2011; Cantiani et al., 2016; Knoth et al., 2018; Côté et al., 2020), we hypothesized that if brain overgrowth is associated with neurological abnormalities, differences between groups would be observed in these responses. More specifically, some studies have shown that macrocephaly, intellectual disability and autism spectrum disorder are associated with a decrease in habituation and novelty detection (Vivanti et al., 2018; Fenckova et al., 2019). Therefore, we hypothesized a decrease in repetition suppression and change detection response in the macrocephalic group in terms of both ERPs components and brain oscillations. Moreover, given that it has been suggested that brain overgrowth is associated with changes in brain organization, mainly affecting long-range connectivity (Karbowski, 2003; Lewis et al., 2013), we also hypothesized longer latencies in terms of ERPs and greater decrease in repetition suppression and change detection response in theta oscillations, which are thought to reflect the activity of long-range networks associated with several cognitive processes, in the macrocephalic group (Lopes, and da Silva, 2013; Kikuchi et al., 2015). With respect to the repetition enhancement effect, given that this response has been thought to be complementary to repetition suppression and to participate in memory consolidation, we also hypothesized a decrease in this effect in the macrocephalic group.

Although head circumference measures are significantly correlated with brain volume during the first year of life (Bartholomeusz et al., 2002; Boucher et al., 2018), it has been also argued that additional brain-morphological metrics such as the extra-axial fluid volume and the ventricular cerebrospinal fluid volume may also contribute to explain the enlargements of head circumference observed in developmental disorders such as autism spectrum disorder (Shen et al., 2013; Denier et al., 2022). Hence, with the aim to investigate the specific contribution of brain overgrowth to the overall effect of head circumference on perceptual learning, we added brain volume measures obtained through transfontanellar 3D ultrasound images.

Finally, in order to ensure an equivalent developmental trajectory between groups (control and macrocephalic) we included an adaptive skills measure. This measure allows us to evaluate what an infant typically does and not only what an infant is capable of doing (Harman and Smith-Bonahue, 2010), which is relevant given that in some neurodevelopmental disorders, such as autism, adaptive skills are more sensitive to alterations than general intelligence (Bradshaw et al., 2019). Furthermore, adaptive skills measures have been considered a surrogate of cognitive development frequently used to determine the severity level of a neurodevelopmental disorder (Bradshaw et al., 2018).

## Materials and methods

### Participants

One hundred sixteen healthy infants, aged between 3 and 11 months, were recruited in the Sainte-Justine Mother and Child University Hospital Center in Montreal. A subset of the data was used in a previous study (López-Arango et al., 2021). Families were first contacted in the post-partum department and in the radiology department (when a macrocephaly was already identified by the family doctor) and re-contacted later. Our inclusion criteria were: healthy full-term infants (>37 weeks gestation) who successfully passed their hearing screening. Exclusion criteria were: pregnancy or delivery complications, infants admitted to neonatal intensive care, infants born prematurely (<37 weeks gestation), hydrocephaly or other neurological problems, presence of any syndromic entity or other severe illness.

Infants were classified into control and macrocephalic groups based on head circumference (HC) measures and the World Health Organisation (WHO) reference data. The HC was measured as the largest occipital-frontal circumference, and it was obtained during the EEG acquisition visit. Infants with an occipitofrontal head circumference exceeding the 97th percentile were included in the macrocephalic group. A total of 35 (14 females) infants with macrocephaly and 81 (39 females) normocephalic infants were investigated.

We matched macrocephalic and normocephalic infants one-to-one by age and sex and run separately our analyses on this subsample, then we compared them to those obtained with the entire sample. We found the same main results in both samples, excepting the interaction group, presentation and volume obtained in the N450 latency model, which can be explained by the marginal significance (*p* = 0.44) observed with the entire sample and the decrease in statistical power with the subsample. For this reason, we decided to keep our entire sample to increase our statistical power.

The experimental protocol was approved by the ethics, scientific and administrative Committee at the Sainte-Justine’s Hospital Research Center. All experiments were performed in accordance with relevant guidelines and regulations. Parents provided written informed consent on behalf of the participating infants and were informed that they could withdraw their participation in the study at any time. Background information, medical and developmental history were obtained via an in-house questionnaire completed by the parents.

### Brain volume

Brain volume measures were acquired using 3D transfontanellar ultrasound images. Two experienced radiologist physicians (A.D. and R.E-J.) obtained the 3D transfontanellar ultrasound images in the coronal plane and sagittal plane [voxel size (1 × 1 × 1) mm] with a Philips EPIQ 7 system and the X6-1 matrix-array transducer. Brain volume was computed with a geometric-based method using a 3D ellipsoid estimation technique developed by Boucher et al. (2018).

### Experimental design

The EEG paradigm consisted of a modified version of the auditory task from Basirat and colleagues (Basirat et al., 2014) to investigate repetition effects and change detection processes involved in the learning of a standard sequence of vowels including a local deviant (/a//a//a//i/).

In the task, 80 standard trials (/a//a//a//i/) (80%) were interspersed by 16 global deviant trials (/a/a/a/a/) (20%). A deviant trial was always followed by a standard trial.

**FIGURE 1:**
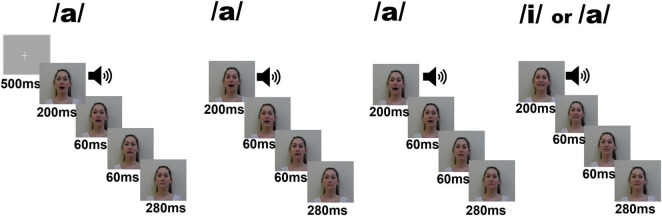
Schematic description of the experimental electrophysiological task. One trial consisted of a sequence of three times the vowel/a/followed by a standard/i/(80 times) or a deviant/a/(16 times). Auditory presentations were supported by visual images (faces pronouncing the vowels). Auditory stimuli were presented during the first 200 ms of each sequence.

In order to maximize attention, auditory stimuli were matched with pictures of a female or male face articulating each vowel. Female and male versions of the sequences were alternated. A fixation point was shown at the beginning of the task (500 ms). Sequences were not separated by an intertrial interval. The stimulus consisted of the auditory vowel lasting 200 ms coinciding with 600 ms visual clips showing the articulation of each vowel as illustrated in Figure 1. Photos used for visual clips and voice recording were obtained from the same male actor and female actress. The fundamental frequencies for the female version were 239 Hz for the/a/vowel and 270 Hz for the/i/vowel. The fundamental frequencies for the male version were 78 Hz for the/a/vowel and 90 Hz for the/i/vowel.

Auditory stimuli were presented at comfortable sound intensity (70 db) via two speakers placed at 30 cm from the infants’ ears. Visual stimuli were presented on a Tobii T120 Eye Tracking screen with 1,024 × 1,280-pixel resolution. Infants were seated on their parent’s lap 60 cm in front of the monitor, in a dark soundproof Faraday cage. Infant gaze was monitored during the experiment using a Tobii T120 eye tracker system. A research assistant remained in the room to keep the infant comfortable and to encourage the infant to look at the screen when necessary.

### Behavioral measure

Adaptive skills were evaluated via the Adaptative Behavioral Assessment System-Second Edition (ABAS-II), parent form (0–5 years old, French-Canadian version) (Oakland and Harrison, 2011), providing composite scores for three adaptive functioning domains (conceptual, social and practical) and a global score [General Adaptative Composite Score (GAC)]. The ABAS-II has been shown to be a reliable and valid instrument for assessing adaptive skills in typical and clinical populations (Oakland and Harrison, 2011; Waisbren et al., 2015; Yarnell et al., 2015; Kirchner et al., 2016).

### Electroencephalograms data acquisition and pre-processing

Continuous EEG was recorded using a 128 electrodes dense array system (124-channels in the infants’ design) (Electrical Geodesics System Inc., Eugene, OR, United States) with impedances kept below 40 kΩ (Turker, 1993) in all electrodes. Signal was acquired at 1,000 Hz sampling rate and referenced online to vertex (Cz). An online bandpass (anti-aliasing) filter of 0.1–500 Hz (Nyquist frequency) was applied. Signals were acquired and stored on a G4 Macintosh computer using NetStation EEG Software (Version 4.5.4).

Pre-processing was performed using MATLAB (R2017b) (The Mathworks Inc., Natick, MA) and the EEGlab toolbox (version 14_1_1b) (Delorme and Makeig, 2004). A high-pass band filter (0.5 Hz) and a “notch” filter (60 Hz) were applied off-line. Twenty-four electrodes containing muscular artefacts placed around the neck and face were excluded (E1, E14, E17, E21, E32, E38, E43, E44, E48, E49, E56, E63, E68, E73, E81, E88, E94, E99, E107, E113, E114, E119, E120, E121). The remaining noisy electrodes were removed using a semi-automatic procedure: electrodes with a total standard deviation higher than 200 μV or lower than 2 μV were automatically removed; electrodes with sporadic behavior were manually removed during subsequent visual inspection. Data were re-referenced to the average reference. Ocular artifacts (eye movement artifacts and blinks) were removed through Independent Component Analysis (ICA, runica algorithm).

Epochs consisting of 3,500 ms were obtained from the continuous EEG data (−1,000 to 2,500 ms relative to the onset of the first vowel in each trial). With the aim to assure an equal number of artifact-free segments for each stimulus of a sequence, artifact rejection was performed per trial. Visual inspection of the segmented data (−1,000 to 2,500 ms relative to the onset of the first vowel in each trial) was performed to manually reject epochs with significant artifacts. After this procedure, the mean number of artifact-free trials available for analysis was 40.7 (control group) (SD = 14.7) and 43.6 (macrocephalic group) (SD = 12.7) for each/a/stimulus of the/a//a//a/sequence and 35.8 (control group) (SD = 12.9) and 36.6 (macrocephalic group) (SD = 10.8) for the local deviant/i/. Repetition effects and change detection response were assessed only with respect to the standard sequence (/a//a//a//i/).

### Time-frequency analysis

A time-frequency approach was used to track spectral energy variation associated with each stimulus presentation over time.

Our analyses were focused on auditory processing, selecting central and frontal electrodes based on previous literature showing that auditory responses are centrally and frontally distributed and that maturational changes in auditory repetition suppression and change detection responses are mainly observed in these regions (Dehaene-Lambertz and Dehaene, 1994; Doeller et al., 2003; He et al., 2009a; Chen et al., 2016; Emberson et al., 2017). We also included right and left frontal regions to investigate possible maturational changes in lateralization (Dehaene-Lambertz and Dehaene, 1994; He et al., 2009a). Four regions of interest (ROI) were considered for analysis: central (5 channels: E7, E31, E55, E80, E106), frontocentral (5 channels:E5, E6, E12, E13, E112), left frontal (6 channels: E19, E20, E23, E24, E27, E28), and right frontal (6 channels: E3, E4, E117, E118, E123, E124) (see Supplementary Figure 1 for an illustration of our ROIs on the Geodesics 128 electrode net). For the channels of each defined ROI, a mean spectral value was computed with MATLAB.

Epochs from 0 to 600 ms for each stimulus of the standard sequence/a//a//a//i/were obtained for time-frequency decomposition. To avoid overlapping between segments and extend our signal segments ensuring an optimal number of points to apply the Morlet’s wavelet transform, a padding technique was performed, in which the first spectral power value of each segment was added for the 600 ms period before and the last spectral power value for the 600 ms after the segment, thus increasing its length (1,800 ms).

A complex Gaussian Morlet’s wavelet transform was performed to obtain time-frequency power maps for each stimulus presentation (see Supplementary Equation 1 for the specific wavelet convolution expression). For the time frequency resolution, we computed 200 frequency points for the range 3–125 Hz that were logarithmically spaced and for the time resolution we computed 400 time slots which gave us a temporal resolution of approximately 2 ms. Thus, an Event Related Spectral Perturbation (ERSP) plot was obtained by stimulus presentation. ERSP shows changes in the EEG spectrum induced by the stimuli presentation at each frequency (Makeig, 1993). ERSP were computed for each trial, and then averaged across trials, using the amplitude and phase given by Morlet’s wavelet transformation (see Supplementary Equation 2 for the equation used for obtaining the ERSP plots). ERSP plots show mean log deviations from baseline power, averaged across participants. A grand mean was computed across subjects and for each ROI.

Baseline was obtained by computing mean spectral power of all four stimulus presentations of the standard sequence at each frequency band (600 ms ERSP plots). Then, the baseline was subtracted from each presentation time-frequency plot.

Inter-trial coherence values (ITC), analogous to phase locking values (PLVs), which measure how consistently the phase at different frequency bands locks to stimulation presented across trials (Tallon-Baudry et al., 1996; Tallon-Baudry and Bertrand, 1999), were also obtained. ITC is reported between 0 and 1 with 0 indicating random phase across trials and 1 perfect intertrial phase alignment of the neural oscillations. ITC was computed for each trial, and then averaged across trials. Finally, a grand mean was computed across subjects and for each ROI.

**FIGURE 2:**
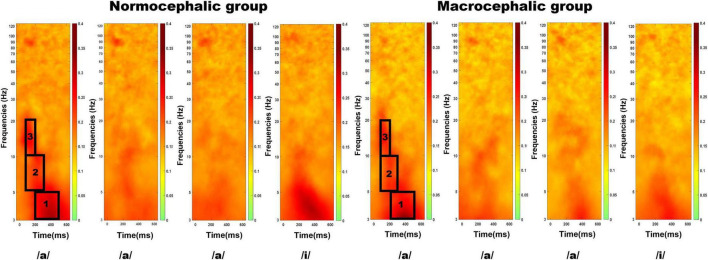
Central region: Inter-trial phase coherence across the standard sequence/a/a/a/i/by group. The *x*-axis represents time, while the *y*-axis displays frequency. Black squares are showing the selected time-frequency windows: (1) 3–5 Hz (200–500 ms); (2) 5–10 Hz (100–300 ms), and (3)10–20 Hz (100–200 ms).

Grand mean ITC plots of the central region, where the auditory response is usually strongest, were used to identify time-frequency windows (TFWs) of interest (see Figure 2 for an illustration of our time-frequency windows). We used ITC plots to define our TFWs of interest since it takes evoked responses into consideration, meaning brain activity that is phase-locked to the stimulus onset. All our ROIs showed similar TFWs at the initial response (see Supplementary Figures 2–4 for TFWs distribution in all our ROIs). Since the boundaries of the various EEG frequency bands change with age, we selected frequency bands that were adequate across the age range studied based on previously published articles (Stroganova and Orekhova, 2007; Saby and Marshall, 2012). As a result, three TFWs were identified: 1. 3–5 Hz (200–500 ms), 2. 5–10 Hz (100–300 ms), and 3.10–20 Hz (100–200 ms) (see Supplementary Figure 5 for an illustration of changes in spectral power across the standard sequence by group).

### Event-related brain potential analysis

Pretreated standard sequences (3,500 ms epochs) were segmented into 800 ms epochs from 200 ms before stimulus onset (baseline) to 600 ms after the onset of each vowel. Segmented epochs were averaged independently for each stimulus presentation after baseline corrections (−200 to 0 ms) over ROIs (central, frontocentral, left frontal, and right frontal).

Peaks in responses to standard and local deviants were identified from the grand average waveform and from the individual ERPs (see Supplementary Figures 8, 9 for ERP waveforms and Supplementary Figures 6, 7 for topographic maps by component). A manual detection method based on developmental literature was applied to analyze ERP waveforms (Kushnerenko et al., 2002; Wunderlich and Cone-Wesson, 2006; Choudhury and Benasich, 2011; Chen et al., 2016). Individual P150 components were defined as highest positive peak within 50–250 ms after stimulus onset in the waveform, N250 responses as the lowest negative peak within 150–350 ms, P350 as the highest positive peak within 250–350 ms and N450 as the lowest negative peak within 300–550 ms. Amplitudes were defined in terms of peak-to-peak measures between components (P150/N250, N250/P350, P350/N450) to attenuate the effect of baseline amplitude variations. When peak identification was doubtful, responses from all ROI were compared, and the response was compared to the grand mean averages. Repetition effects and change detection response were analyzed through the variation of these peak-to-peak amplitudes across presentations (/a//a//a//i/) [see Supplementary Figure 8 for an illustration of Grand average ERPs waveforms for the first, second and third/a/presentation and Supplementary Figure 9 for an illustration of the Grand Average ERPs waveforms per condition (standard vs. deviant)].

Latencies were defined from stimulus onset to the highest amplitude for each component.

After preprocessing, no filter was applied for ERPs analysis.

### Statistical analysis

A linear mixed model (LMM) approach was used to characterize the pattern of response across presentations of the standard sequence (/a//a//a//i/) (IBM SPSS Statistics version 24). This approach also allowed us to deal with unbalance data sets and with missing values (Field, 2009; West, 2009).

A baseline model was built by time-frequency window (TFW) and by component to investigate changes in brain response (spectral power, phase coherence, amplitude, and latency) with regard to stimulus presentation. Brain response was considered our dependent variable, whereas group (normocephalic and macrocephalic), region of interest (ROI) and presentation were placed as fixed effects into the model (2 groups × 4 presentations × 4 ROIs). Interactions between fixed effects were also considered in the model.

Subsequently, random intercept and random slope were sequentially tested to identify our best model fit (Field, 2009; Shek and Ma, 2011). Finally, age, brain volume and GAC score were sequentially added to the model as predictors. We only retained predictors that resulted in a significant increase in model fit compared to a reduced version of the model. A χ^2^-difference test was conducted to assess likelihood ratio tests of the full model and to determine the best model fit (West, 2009). The Holm-Bonferroni method was applied to correct for multiple hypotheses testing and we report significant corrected *p*-values (Holm, 1979; Gaetano, 2018).

With the aim to further investigate if the variance in two processes of interest (repetition effects between the first and the second presentation, and change detection response) can be significantly explained by group, age, brain volume and adaptive skills, we performed hierarchical regression analysis for each response and for each ROI (central, frontocentral, left frontal, and right frontal). We calculated repetition effects as the difference between the response to the second and first presentation (second – first). Change detection response was calculated by subtracting the mean standard response (average response to the three/a/presentations) from deviant response (deviant – standard). Group, age, brain volume and adaptive skills were successively added to assess their contribution to the model. Adjusted R^2^ (explanation of variance), incremental explanation of variance, standardized beta values (β) and *p*-values of the change in variance between the models were computed. This procedure was repeated by process (repetition suppression effect and change detection response) and by ROI. Bonferroni corrections for multiple testing were applied by model (α/k, where α is 0.05 and k is the number of predictors by model). Statistical significance was set at *p* < 0.05.

ERPs amplitudes (P150/N250, N250/P350, P350/N450) and latencies (P150, N250, P350, N450), spectral power and ITC were analyzed following the same sequence of analysis (LMM and hierarchical linear regression).

Assumptions of linearity, homoscedasticity, normality and collinearity were previously verified. None of the independent variables (age, GAC score, and brain volume) correlated higher than 0.7 with each other. Tolerance values were higher than 0.3, while variance inflation factors (VIF) were lower than 5 (Stafford et al., 2006).

## Results

### Demographic data

Based on independent samples *t*-tests, there were no statistically significant differences between groups regarding age [*t*(114) = 1.06, *p* = 0.29] or GAC scores *[t(*108) = 0.303, *p* = 0.76]. Head circumference [*t*(114) = 8.11, *p* < 0.0001] and brain volume [*t(*105) = 6.47, *p* < 0.0001] were significantly different between groups (see Table 1 for characteristics of control and macrocephalic infants and Supplementary Table 1 for age distribution by group).

**TABLE 1:** Characteristics of control and macrocephalic infants.

Group	Sex		Total	Age		GAC score	Head circumference	Brain volume
	Male	Female		Mean (SD)	Age range	Mean (SD)	Mean (SD)	Mean (SD)
Macrocephalic	21	14	35	6.54 (2.29)	3–11 months	102.48 (11.34)	46.11 (2.13)	823.36 (102.66)
Control	42	39	81	6.11 (1.89)	3–11 months	101.53 (16.01)	43.15 (1.6)	704.25 (78.94)
Total	63	53	116					

There were six missing values on the GAC score (2 control and 4 macrocephalic) and nine missing values (5 control and 4 macrocephalic) of brain volume measures.

### Electroencephalograms data

#### Total sample repetition effects and change detection response

Our total sample showed significant changes in slope with respect to (/a/) repetitions and the deviant stimuli (/i/). The contribution of age, GAC score and brain volume were evaluated in all our models. Only significant results after *p*-value correction are reported (Holm, 1979; Gaetano, 2018).

#### Repetition effects in spectral power, inter-trial coherence values, and event-related brain potentials

Our total sample showed a repetition suppression effect as measured by spectral power and ITC in all the TFWs. We provide statistical details in the Supplementary Material (see Supplementary Table 4 for estimates of fixed effects for spectral power by TFW and Supplementary Table 7 for estimates of fixed effects for ITC by TFW). In terms of ERPs, the N450 component showed a repetition effect, with a decrease in P350/N450 peak-to-peak amplitude (repetition suppression) and a decrease in latency associated with the second/a/presentation (see Supplementary Table 10 for estimates of fixed effects for peak-to-peak measure and Supplementary Table 13 for estimates of fixed effects for latency by component).

#### Change detection response in spectral power, inter-trial coherence values, and event-related brain potentials

In the total sample, slope increases in power, ITC and ERP amplitude were significant in response to the deviant vowel/i/in all our TFWs and in all our peak-to-peak values (P150/N250, N250/P350, and P350/N450) (see Supplementary Table 3 for best model fit statistics by TFW for spectral power, Supplementary Table 6 for best model fit statistics by TFW for ITC and Supplementary Table 9 for best model fit statistics by peak-to-peak measure).

#### Group difference (normocephalic vs. macrocephalic) in repetition effects

(a) **Spectral power**

We did not find group differences in repetition effects in terms of spectral power.

(b) **ITC**

**10–20 Hz (100–200 ms)**

**FIGURE 3:**
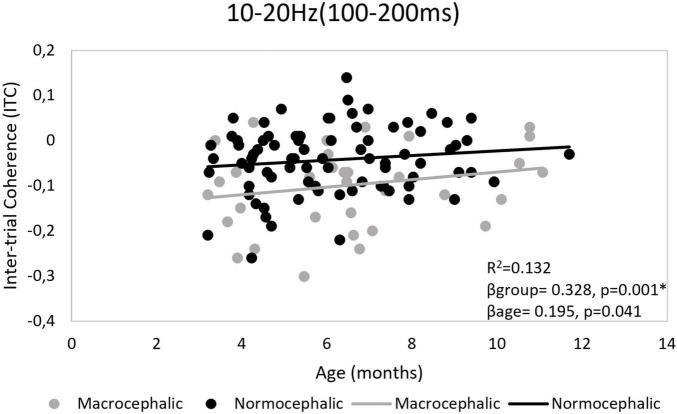
Right frontal region. Ten to twenty Hertz time-frequency window (TFW). Scatter plot illustrating group effect for Inter-trial phase coherence (ITC). Control infants showed less repetition suppression than macrocephalic infants. Repetition suppression was calculated as the difference between the response to the second and first presentation (second – first). *Adjusted *p*-value < 0.0125.

Although a general repetition suppression response as measured by ITC was observed, a differential response pattern by group was revealed by LMM and regression models. LMM showed more repetition suppression in the macrocephalic group compared to the control group [*b* = −0.06, *t*(786.4) = −3.8; *p* < 0.0006*]. Regression models confirmed this result and showed that differences between groups were particularly observed in the right frontal region. In this region, repetition effects were significantly associated with group [*F*(2, 99) = 7.5, *p* = 0.001, *R*^2^ = 0.132], showing less repetition suppression in the normocephalic group compared to the macrocephalic group (β*group* = 0.328, *p* = 0.001*; β*age* = 0.195, *p* = 0.041). Bonferroni correction by model. *Adjusted *p*-value < 0.0125 (see Figure 3).

(c) **ERPs. Amplitude**

**N250/P350 peak-to-peak amplitude**

LMM showed a significant interaction between group, presentation and GAC score in N250/P350 peak-to-peak amplitude. In this case, macrocephalic infants with higher GAC score showed a repetition enhancement effect [*b* = 0.188, *t*(370.96) = 2.03; *p* = 0.043*]. It is worth mentioning that for this measure we did not observe a repetition suppression effect but an increase in response across presentations, reaching its maximum amplitude with the deviant/i/vowel (quadratic slope) [*b* = 0.53, *t*(557.92) = 7.96; *p* < 0.0004]. In addition, although not statistically significant, less repetition enhancement was observed in the macrocephalic group [*b* = −18.58, *t*(371.12) = −2.02; *p* = 0.086] (see Supplementary Table 8 for descriptive statistics for peak-to-peak measure by group and Supplementary Table 10 for estimates of fixed effects by peak-to-peak measure).

(d) **ERPs. Latency**

**N450 latency**

Although we observed a significant reduction in N450 component latency associated with repetition, the interaction among group, presentation and volume shows longer latencies in normocephalic infants with larger brain volume [*b* = 0.263, *t*(192.1) = 2.29; *p* = 0.044*]. This effect is attenuated in normocephalic infants with higher GAC scores [*b* = −0.0026, *t*(169.42) = −2.57; *p* = 0.044*] (see Supplementary Table 13 for estimates of fixed effects by component, Supplementary Table 12 for best fit model by ERP component for latency and Supplementary Table 11 for descriptive statistics for latency by ERP component).

#### Group difference (normocephalic vs. macrocephalic) in change detection response

(a) **Spectral power**

**10–20 Hz (100–200 ms)**

**FIGURE 4:**
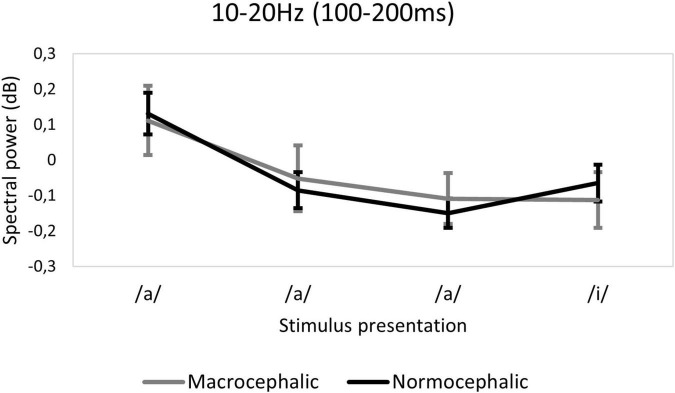
Ten to twenty Hertz time-frequency window (TFW). Spectral power. Plot demonstrating the pattern of response across presentations of the standard sequence by group. Error bars indicate 95% confidence intervals. dB, decibels.

**FIGURE 5:**
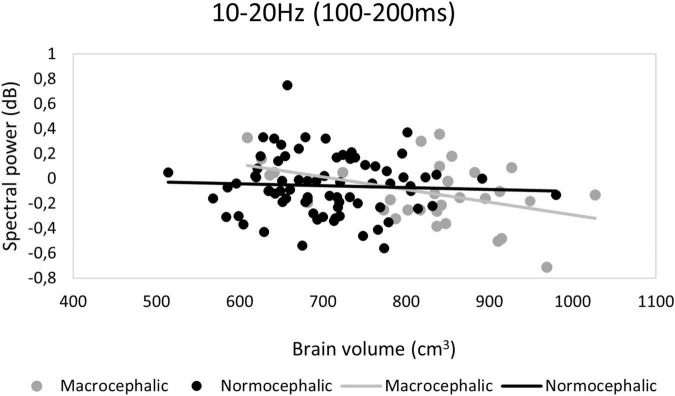
Ten to twenty Hertz time-frequency window (TFW). Change detection response: Scatter plot demonstrating the interaction between group, brain volume and quadratic slope. A decrease in change detection response was associated with increasing brain volume only in the macrocephalic group (all ROIs) in terms of spectral power. dB, decibels.

**FIGURE 6:**
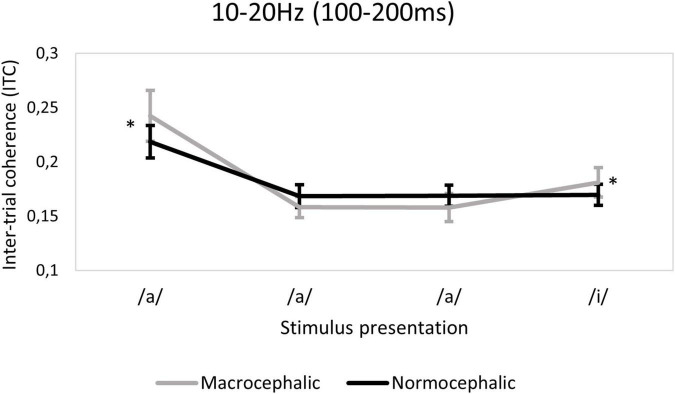
Ten to twenty Hertz time-frequency window (TFW). Plot demonstrating the group effect, showing the macrocephalic group to have a greater response to the first/a/presentation (corrected *p* < 0.0006*) compared to the normocephalic group. The plot also illustrates the interaction between group and quadratic slope, showing only macrocephalic infants to have a change detection response associated with the last/i/stimulus (corrected *p* < 0.0006*). Error bars indicate 95% confidence intervals.

We found that the spectral power response pattern across presentations by group in the 10–20 Hz TFW was opposite to the pattern observed in terms of ITC (see Figures 4, 6). In this TFW, a repetition suppression effect and a change detection response were observed in spectral power (see Supplementary Table 2 for descriptive statistics for spectral power by group). However, a significant interaction between group, quadratic slope and brain volume revealed that macrocephalic infants with larger brain volume showed less change detection response [*F*(2, 682.11) = 5.41; *p* = 0.015*] (see Figure 5).

(b) **ITC**

**10–20 Hz (100–200 ms)**

A main effect of group was found in the 10–20 Hz TFW in terms of ITC [*F*(1, 874.51) = 13.5; *p* < 0.0006*]. More precisely, in macrocephalic infants, a higher ITC at the initial/a/presentation was observed [*b* = 0.07, *t*(874.51) = 3.73; *p* < 0.0006*] compared to the normocephalic group. In addition, a significant interaction between group and quadratic slope suggests higher ITC associated with the deviant (/i/) in the macrocephalic group [*F*(1, 801.53) = 13.9; *p* < 0.0006*] (see Figure 6 and Supplementary Table 5 for descriptive statistics for ITC by group).

(c) **ERPs. Amplitude**

We did not find a group difference in change detection response in terms of ERPs amplitude.

(d) **ERPs. Latency**

We did not find a group difference in change detection response in terms of ERPs latency.

## Discussion

We aimed to investigate if repetition effects and change detection response as measured through EEG differ between normocephalic and macrocephalic infants during the first year of life. We hypothesized a decrease in the repetition suppression effect and the change detection response in the macrocephalic group. We also hypothesized longer ERPs latencies in the macrocephalic group and greater differences between groups in the theta brain oscillations. In fact, we found robust repetition suppression in all our TFWs and in the N450 component (peak-to-peak amplitude and latency) in both infant groups. However, normocephalic infants with larger brain volume showed longer latencies in the N450 component associated with the second/a/presentation, whereas the macrocephalic group showed peculiarities in the 10–20 Hz TFW in both repetition effects and change detection response.

In fact, macrocephalic infants showed more repetition suppression as measured by ITC in the 10–20 Hz TFW. The greater repetition suppression in the 10–20 Hz TFW is seemingly caused by the increased ITC to the initial standard stimulus of a sequence in the macrocephalic group.

Similarly, macrocephalic infants showed augmented ITC in the 10–20 Hz frequency range in response to the deviant stimuli. On the other hand, they showed a significant decrease in spectral power associated to change detection in the same frequency range. This decrease was negatively correlated with brain volume in macrocephalic infants, indicating that lower spectral power in response to the deviant vowel is associated with greater brain volume. These findings could be explained by the fact that spectral power and ITC do not reflect the same neural activity. Whereas ITC reflects phase-locked neural activity consistent across stimulus trials, spectral power reflects not only phase-locked but also induced neural processing non-phase-locked to the stimulus but generated in response to the stimulus presentation (Bidelman, 2015; Mahmud et al., 2021). Whether the peculiarities found in the macrocephalic group in terms of ITC and spectral power in the 10–20 Hz frequency range are caused by bottom-up or top-down processes, is unclear. However, it has been suggested that changes in phase locking synchronization may mainly reflect bottom-up processing, whereas power changes in oscillatory activity may reflect the interaction between the processing of sensory information as well as ongoing neuronal activity, including top-down processing (Busch et al., 2006; Schneider et al., 2008; Chen et al., 2012).

A likely explanation of the increased synchronization (ITC) of beta activity would be that macrocephalic infants processed the initial stimulus of the standard sequence (/a/a/a/i/) as more salient and they reacted with a higher level of alertness or cortical activation (Gola et al., 2012; Kamiński et al., 2012). Moreover, the fact that the greater repetition suppression effect was only observed in the right frontal region may support a greater involvement of the right attentional network in the initial response, possibly confirming higher cortical activation in the macrocephalic group. On the other hand, greater amplitude and sharper responses to sensory stimulation have been demonstrated in neurodevelopmental disorder populations such as the Fragile X syndrome (Knoth et al., 2014; Côté et al., 2020). Whether this pattern of response is predictive of later cognitive outcomes remains to be studied.

Regarding the change detection response, greater chance detection response in the 10–20 Hz frequency range has already been reported in infants and adults when auditory paradigms are used (Haenschel et al., 2000; Isler et al., 2012). In our study, the greater change detection response as measured by ITC in macrocephalic infants may suggest that they have not learned the frequent changes (expected change rate: 80% for the/a/a/a/i/sequence), contrary to normocephalic infants (Deguire et al., 2022).

The decrease in spectral power in the 10–20 Hz TFW in response to change detection in macrocephalic infants may reflect a decrease in other perceptual or cognitive processes, maybe top-down processing recruited by our task. Some studies have proposed that an increase in frontal alpha and beta oscillations may reflect inhibitory functions that facilitate working memory processing (Schmidt et al., 2019). In accordance with this hypothesis, it is possible that although macrocephalic infants synchronized neural ensembles to process the deviant stimulus, they showed less activation of the inhibitory functions associated with beta oscillations to facilitate working memory processing.

Infants in the macrocephalic group also processed the repeated stimulus differently in terms of ERPs. We found a N250/P350 peak-to-peak repetition enhancement effect that significantly increased in macrocephalic infants with higher GAC scores, suggesting that adaptive skills may be associated with brain responses and that higher adaptive skills may foster the processing involved in these brain responses. In infants, the second positivity has been thought to reflect stimulus awareness and auditory memory recognition (De Haan, 2007; Choudhury and Benasich, 2011). Additionally, the repetition enhancement effect has been proposed as an index of acoustic memory trace formation (Recasens et al., 2015). Thus, our results may suggest that macrocephalic infants with higher adaptive skills, compared to macrocephalic infants with lower adaptive skills, are able to recognize and encode the second/a/stimulus in a more similar way to normocephalic infants maybe not individually but as part of the sequence/a/a/a/, thus showing the same N250/P350 peak-to-peak repetition enhancement effect observed in the normocephalic group.

Regarding the N450 component, a decrease in latency associated with repetition was observed. This pattern is thought to reflect a repetition priming effect and more efficient stimulus processing (Gotts et al., 2012); however, normocephalic infants with larger brain volume showed longer latencies associated with the second presentation, suggesting that brain overgrowth delays stimulus processing associated with the N450 component. Furthermore, adaptive skills seem to modulate N450 latency, given that a decrease in latency associated with repetition was also observed in normocephalic infants with higher GAC scores. These findings suggest that the N450 repetition effect as measured by latency is sensitive to both, brain volume and adaptive skills.

On the contrary, change detection response as measured by ERPs (amplitude and latency) were not sensitive to macrocephaly, which is in line with previous research showing that brain oscillations constitute a more sensitive marker of the brain’s response to change detection than ERP measures in infants (Isler et al., 2012).

Finally, taking into consideration the audio-visual nature of our task, we cannot discard that the differences observed between the groups could be facilitated or enhanced by the bimodality of the task. Indeed, it has been shown that a bimodal presentation enhances auditory processing (Bahrick and Lickliter, 2000; Bahrick et al., 2002; Reynolds et al., 2014). Further, the audio-visual presentation could also play a role in the involvement of beta oscillations (10–20 Hz) as a sensitive marker to macrocephaly, given that this frequency range has also been associated with multisensorial integration (von Stein et al., 1999). Hence, our results may suggest that macrocephalic infants have more audio-visual attention processes deployed in multimodal tasks.

### Limitations

Our study provides first evidence that brain overgrowth, a subtype of macrocephaly, present as an isolated trait in otherwise neurotypical infants, may have an impact on sensorial learning processing indexed by repetition effects and change detection response. However, there are some limitations in this study that could be addressed in future research. First, we acknowledge that some participants contributed with a low number of trials, affecting our signal-to-noise ratio. Second, we were unable to analyze the global change detection response because of the reduced number of global deviants. Third, although we obtained significant associations in terms of *p*-value, our effect sizes are small or medium. Fourth, we focused our analysis on specific ROIs, which contributes to reduce spatial effects by averaging channel signals, however, future studies should include a source location method to ensure independence of ERPs sources/components and, to provide more specific information on the ERPs generators. Finally, although we included adaptive skills to compare the general developmental trajectory between groups, future studies should incorporate additional cognitive measures.

## Conclusion

Taken together, our results suggest that macrocephaly may have a significant impact on repetition effects and the change detection response during the first year of life, mainly promoting higher responses to change, although this does not necessarily mean that macrocephalic infants process changing information more efficiently.

Interestingly, our results also suggest that brain oscillations, assessed in terms of spectral power and ITC, are more sensitive than ERPs to brain overgrowth during the first year of life.

In conclusion, further investigation and the follow-up of our cohort will allow us to determine the predictive value of brain overgrowth and the EEG brain response peculiarities during the first year of life to identify developmental trajectories.

## Data availability statement

The raw data supporting the conclusions of this article will be made available by the authors, without undue reservation.

## Ethics statement

The studies involving human participants were reviewed and approved by the Ethics, Scientific and Administrative Committee at the Sainte-Justine’s Hospital Research Center. Written informed consent to participate in this study was provided by the participants’ legal guardian/next of kin. Written informed consent was obtained from the individual(s) for the publication of any potentially identifiable images or data included in this article.

## Author contributions

GL-A designed the study, acquired, analyzed, and interpreted the data, and drafted the initial manuscript. FD contributed in data collection and analysis. KA and M-AB contributed to the analytic tools. IK conceptualized and implemented the study and participated in data collection and analysis. RE-J contributed in the data collection. VC designed the study. AD designed the study and contributed in recruitment and data collection. SK designed the study and contributed to the analytic tools. SL conceptualized and designed the study, and interpreted the data. All authors critically revised the manuscript and approved the submitted version.

## Conflict of interest

The authors declare that the research was conducted in the absence of any commercial or financial relationships that could be construed as a potential conflict of interest.

## Publisher’s note

All claims expressed in this article are solely those of the authors and do not necessarily represent those of their affiliated organizations, or those of the publisher, the editors and the reviewers. Any product that may be evaluated in this article, or claim that may be made by its manufacturer, is not guaranteed or endorsed by the publisher.
