# Supplementary material for: Impact of brain overgrowth on sensorial learning processing during the first year of life
Source: Front Hum Neurosci. 2022 Jul 19;16:928543. doi: 10.3389/fnhum.2022.928543 (PMC9344916; doi:10.3389/fnhum.2022.928543)
Supplement: Supplementary file 1 [file Data_Sheet_1.PDF]

Supplementary Material

1 Supplementary Figures and Tables

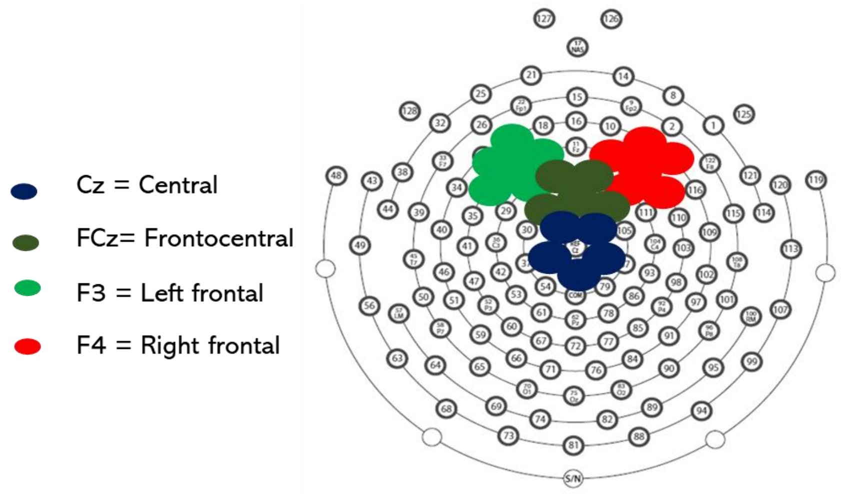

Supplementary Figure S1. Regions of interest (ROIs) on the Geodesics 128 electrode net: Central, frontocentral, left frontal and right frontal.

Supplementary Table S1. Age distribution by group.

	Age range (months)			
**Group**	**3-5**	**6-8**	**9-11**	**Total**
**Macrocephalic**	13	16	6	35
**Normocephalic**	41	32	8	81
**Total**	54	48	14	116

Supplementary Table S2. Descriptive statistics (mean and standard deviation) for spectral power (db) by group, TFW and ROI per stimulus of the standard sequence /a//a//a//i/.

		**3-5Hz(100-300ms) TFW**			
	Group	/a/	/a/	/a/	/i/
**Central**	Macrocephalic	0.087(0.38)	-0.35(0.43)	-0.169(0.41)	0.269(0.35)
	Control	0.040(0.56)	-0.26(0.44)	-0.133(0.52)	0.150(0.50)
**Frontocentral**	Macrocephalic	0.145(0.38)	-0.294(0.50)	-0.120(0.43)	0.109(0.38)
	Control	0.054(0.49)	-0.300(0.53)	-0.169(0.53)	0.203(0.53)
**Left frontal**	Macrocephalic	0.118(0.51)	-0.317(0.55)	-0.208(0.45)	0.212(0.45)
	Control	0.066(0.51)	-0.401(0.57)	-0.211(0.53)	0.326(0.48)
**Right frontal**	Macrocephalic	0.127(0.40)	-0.316(0.57)	-0.169(0.57)	0.154(0.57)
	Control	-0.013(0.50)	-0.277(0.50)	-0.074(0.52)	0.159(0.55)

		**5-10Hz(100-300ms) TFW**			
	Group	/a/	/a/	/a/	/i/
**Central**	Macrocephalic	0.023(0.45)	-0.164(0.43)	-0.085(0.40)	0.063(0.43)
	Control	-0.40(0.46)	-0.124(0.46)	-0.115(0.42)	0.094(0.49)
**Frontocentral**	Macrocephalic	0.099(0.41)	-0.132(0.48)	-0.132(0.46)	0.005(0.41)
	Control	0.019(0.43)	-0.157(0.48)	-0.135(0.39)	0.090(0.43)
**Left frontal**	Macrocephalic	0.024(0.45)	-0.147(0.38)	-0.153(0.40)	0.120(0.38)
	Control	0.052(0.42)	-0.195(0.43)	-0.104(0.41)	0.075(0.42)
**Right frontal**	Macrocephalic	0.096(0.37)	-0.105(0.43)	-0.191(0.39)	0.038(0.48)
	Control	0.049(0.48)	-0.173(0.49)	-0.123(0.42)	0.057(0.46)

		**10-20Hz(100-200ms) TFW**			
	Group	/a/	/a/	/a/	/i/
**Central**	Macrocephalic	0.086(0.44)	-0.060(0.40)	-0.081(0.34)	-0.110(0.32)
	Control	0.124(0.39)	-0.074(0.37)	-0.146(0.32)	-0.065(0.36)
**Frontocentral**	Macrocephalic	0.124(0.35)	0.002(0.40)	-0.121(0.36)	-0.167(0.35)
	Control	0.150(.042)	-0.092(0.40)	-0.183(0.34)	-0.056(0.37)
**Left frontal**	Macrocephalic	0.063(0.40)	-0.072(0.33)	-0.087(0.29)	-0.62(0.35)
	Control	0.082(0.33)	-0.070(0.36)	-0.112(0.34)	-0.053(0.35)
**Right frontal**	Macrocephalic	0.174(0.41)	-0.078(0.42)	-0.147(0.32)	-0.112(0.39)
	Control	0.171(0.37)	-0.103(0.38)	-0.156(0.37)	-0.086(0.37)

Supplementary Table S3. Best model fit statistics by time-frequency window. Spectral power (dB). LMM (2 groups x 4 presentations x 4 ROI).

**TFW**	**Fixed effects, quadratic slope, GAC score and brain volume as predictors**	**Effect**	**df**	**F**	**p value [1]**
3-5 Hz 200-500ms	$\chi2(1,N=116)=97.92$, $p<.0001$	Presentation	(1, 547.72)	24.6	$p<0.0002$*
		Quadratic slope	(1, 778.39)	143.82	$p<0.0002$*
5-10Hz 100-300ms	$\chi2(1,N=116)=75.33$, $p<0.0001$	Presentation	(1, 561.73)	5.97	$p=0.015$*
		Quadratic slope	(1, 767.14	47.13	$p<0.0002$*
10-20Hz 100-200ms	$\chi2(3,N=116)= 53.01$, $p<0.0001$	Intercept	(1, 737.89)	5.8	$p=0.030$*
		Presentation	(1, 629.93)	9.05	$p=0.012$*
		Quadratic slope	(1,805.23)	11.82	$p=0.005$*
		Groupe(Macrocephalic)* Quadratic slope*Brain Volume	(2,682.11)	5.4	$p=0.015$*

1. Holm-Bonferroni method. *p<.05

Note: (df) degree of freedom

Supplementary Table S4. Estimates of fixed effects by time-frequency window (TFW). Spectral power. LMM (2 groups x 4 presentations x 4 ROIs).

TFW	Effect	b (SE)	corrected p value*
3-5Hz	Presentation	-0.798(0.189)	*p*<0.0002*
	Quadratic slope	0.185(0.15)	*p*<0.0002*
5-10Hz	Presentation	-0.434(0.17)	*p*=0.009*
	Quadratic slope	0.091(0.013)	*p*<0.0002*
10-20Hz	Intercept	1.37(0.57)	*p*=0.032*
	Presentation	-1.14(0.55)	*p*=0.038*
	Quadratic slope	0.24(0.09)	*p*=0.027*
	Group (Macrocephalic) *Quadratic slope* Brain volume	-0.00044(0.00014)	*p*=0.004*

1. Holm-Bonferroni method. **p*<.05

Note: (SE) Standard error.

Supplementary Table S5. Descriptive statistics (mean and standard deviation) for ITC by group, TFW and ROI per stimulus of the standard sequence /a//a//a//i/.

		3-5Hz(100-300ms) TFW			
	Group	/a/	/a/	/a/	/i/
**Central**	Macrocephalic	0.296(0.10)	0.188(0.07)	0.234(0.09)	0.272(0.11)
	Control	0.270(0.13)	0.203(0.08)	0.190(0.07)	0.254(0.09)
**Frontocentral**	Macrocephalic	0.289(0.12)	0.220(0.08)	0.220(0.08)	0.266(0.10)
	Control	0.280(0.12)	0.204(0.09)	0.195(0.08)	0.248(0.10)
**Left frontal**	Macrocephalic	0.283(0.10)	0.224(0.07)	0.201(0.08)	0.227(0.09)
	Control	0.267(0.11)	0.206(0.08)	0.197(0.07)	0.235(0.10)
**Right frontal**	Macrocephalic	0.260(0.12)	0.213(0.07)	0.211(0.08)	0.257(0.10)
	Control	0.266(0.11)	0.214(0.09)	0.197(0.08)	0.247(0.11)

		5-10Hz(100-300ms) TFW			
	Group	/a/	/a/	/a/	/i/
**Central**	Macrocephalic	0.228(0.09)	0.188(0.06)	0.177(0.07)	0.217(0.09)
	Control	0.219(0.09)	0.194(0.07)	0.167(0.07)	0.195(0.08)
**Frontocentral**	Macrocephalic	0.242(0.09)	0.209(0.07)	0.203(0.08)	0.222(0.11)
	Control	0.237(0.10)	0.204(0.08)	0.199(0.07)	0.215(0.09)
**Left frontal**	Macrocephalic	0.241(.11)	0.197(0.08)	0.195(0.08)	0.216(0.10)
	Control	0.224(0.09)	0.201(0.08)	0.200(0.09)	0.205(0.08)
**Right frontal**	Macrocephalic	0.237(0.08)	0.200(0.07)	0.198(0.06)	0.240(0.10)
	Control	0.238(0.10)	0.212(0.09)	0.206(0.07)	0.221(0.09)

		10-20Hz(100-200ms) TFW			
	Group	/a/	/a/	/a/	/i/
**Central**	Macrocephalic	0.248(0.09)	0.157(0.40)	0.163(0.05)	0.173(0.06)
	Control	0.222(0.08)	0.170(0.06)	0.173(0.06)	0.161(0.06)
**Frontocentral**	Macrocephalic	0.256(0.08)	0.163(0.04)	0.162(0.05)	0.181(0.05)
	Control	0.229(0.09)	0.171(0.06)	0.172(0.05)	0.179(0.06)
**Left frontal**	Macrocephalic	0.213(0.06)	0.163(0.05)	0.161(0.05)	0.173(0.06)
	Control	0.210(0.08)	0.162(0.06)	0.166(0.06)	0.172(0.06)
**Right frontal**	Macrocephalic	0.249(0.09)	0.150(0.05)	0.151(0.04)	0.196(0.07)
	Control	0.213(0.07)	0.170(0.06)	0.165(0.06)	0.168(0.05)

Supplementary Table S6. Best model fit statistics by TFW (ITC). LMM (2 groups x 4 presentations x 4 ROI).

Frequency band	Best fit model.	Effect	F (df)	corrected p value [1]
3-5 Hz 200-500ms	Random intercept and quadratic slope. [$\chi 2$(1,N=116) = 112.95, *p*<0.0001]	Intercept	*F*(1, 714.99)= 671.66	*p*<0.0003*
		Presentation	*F*(1, 606.51)= 131.99	*p*<0.0003*
		Quadratic slope	*F*(1,624.09)=124.36	*p*<0.0003*
5-10Hz 100-300ms	Random intercept, random slope and quadratic slope. [$\chi 2$(1, N=116) = 37.97, *p*<0.0001]	Intercept	*F*(1,684.88)=511.35	*p*<0.0003*
		Presentation	*F*(1,575.25)=43.47	*p*<0.0003*
		Quadratic slope	*F*(1, 590.51)=39.03	*p*<0.0003*
10-20Hz 100-200ms	Random intercept and quadratic slope. [$\chi 2$(1, N=116)= 114.41, *p*<0.0001]	Intercept	*F*(1, 777)=1059.71	*p*<0.0006*
		Presentation	*F*(1, 659.97)=172.8	p<0.0006*
		Quadratic slope	*F*(1, 670.84)=129.02	*p*<0.0006*
		Group	*F*(1, 874.51)=13.5	p<0.0006*
		Group*Presentation	*F*(1, 786.4)=14.08	p<0.0006*
		Group*Quadratic Slope	*F*(1, 801.53)=13.9	p<0.0006*

1. Holm-Bonferroni method. *p<.05

Note: (df) degree of freedom

Supplementary Table S7. Estimates of fixed effects by TFW (ITC). LMM (2 groups x 4 presentations x 4 ROIs). Group effects were observed in the 5-10Hz and 10-20Hz TFWs.

TFW	Effect	Average (SE)	corrected p value*
3-5Hz	Intercept	0.038(0.015)	*p*<0.0003*
	Presentation	-0.145(0.012)	*p*=0.0003*
	Quadratic Slope	0.028(0.002)	*p*<0.0003*
5-10Hz	Intercept	0.28(0.013)	*p*<0.0003*
	Presentation	-0.074(0.010)	*p*<0.0003*
	Quadratic slope	0.013(0.002)	*p*<0.0003*
10-20Hz	Intercept	0.29(0.011)	*p*<0.0006*
	Presentation	- 0.08(0.1)	p<0.0006*
	Quadratic slope	0.01(0.002)	p<0.0006*
	Group= Macrocephaly	0.07(0.02)	p<0.0006*
	Group=Macrocephaly*Presentation	-0.06(0.02)	p<0.0006*
	Group= Macrocephaly* Quadratic slope	0.012(0.003)	p<0.0006*

1. Holm-Bonferroni method. *p<.05

Note: (SE) Standard error.

Supplementary Table S8. Descriptive statistics (mean and standard deviation) for amplitude (μV) by group, peak-to-peak measure and ROI per stimulus of the standard sequence /a//a//a//i/.

		P150/N250			
	Group	/a/	/a/	/a/	/i/
**Central**	Macrocephalic	-2.93(2.51)	-1.69(1.09)	-2.58(1.44)	-3.53(1.94)
	Normocephalic	-4.14(2.49)	-2.89(1.88)	-3.85(2.91)	-4.27(3.03)
**Frontocentral**	Macrocephalic	-3.22(2.20)	-2.19(1.56)	-2.91(1.54)	-3.01(1.94)
	Normocephalic	-3.63(3.13)	-3.00(2.30)	-3.82(2.67)	-3.49(2.40)
**Left frontal**	Macrocephalic	-3.90(2.52)	-2.95(2.13)	-4.13(2.48)	-3.80(1.96)
	Normocephalic	-3.84(2.97)	-3.97(2.88)	-4.66(3.50)	-3.89(2.72)
**Right frontal**	Macrocephalic	-3.96(1.98)	-3.20(2.04)	-3.67(1.76)	-4.57(2.22)
	Normocephalic	-4.16(2.03)	-4.27(2.59)	-4.85(2.87)	-4.69(2.63)

		N250/P350			
	Group	/a/	/a/	/a/	/i/
**Central**	Macrocephalic	3.06(1.80)	4.20(2.41)	3.00(1.33)	4.83(2.68)
	Normocephalic	3.78(3.03)	4.73(3.32)	3.34(2.48)	5.02(2.90)
**Frontocentral**	Macrocephalic	3.57(2.25)	4.27(2.44)	3.74(1.93)	5.90(3.15)
	Normocephalic	4.53(2.60)	5.03(2.67)	3.96(1.88)	7.17(3.78)
**Left frontal**	Macrocephalic	5.09(2.74)	5.17(3.07)	4.44(2.31)	6.52(2.97)
	Normocephalic	5.72(2.85)	5.45(2.67)	5.02(2.58)	7.75(3.33)
**Right frontal**	Macrocephalic	4.33(2.28)	4.94(2.49)	4.55(2.01)	7.25(3.33)
	Normocephalic	5.22(2.96)	5.48(3.20)	4.68(2.96)	7.63(4.02)

		P350/N450			
	Group	/a/	/a/	/a/	/i/
**Central**	Macrocephalic	-9.82(4.22)	-3.58(2.11)	-5.29(2.74)	-6.69(3.40)
	Normocephalic	-9.33(5.26)	-4.45(2.09)	-5.21(2.75)	-7.01(3.56)
**Frontocentral**	Macrocephalic	-10.36(5.47)	-4.71(2.48)	-5.65(2.88)	-7.00(3.68)
	Normocephalic	-9.54(5.86)	-4.87(2.95)	-5.27(3.34)	-7.41(4.53)
**Left frontal**	Macrocephalic	-10.30(5.29)	-6.46(2.71)	-6.42(2.47)	-8.31(4.23)
	Normocephalic	-10.33(5.48)	-5.95(3.05)	-6.22(3.40)	-9.08(4.76)
**Right frontal**	Macrocephalic	-11.03(5.22)	-6.31(2.50)	-6.66(2.69)	-8.38(4.03)
	Normocephalic	-10.59(5.78)	-6.12(3.15)	-5.90(2.63)	-8.57(4.40)

Supplementary Table S9. Best model fit statistics by peak-to-peak measure (μV). LMM(2Groups x 4 presentations x 4 ROI).

**Peak-to-peak amplitude**	**Best fit model**	**Effect**	***F* (df)**	**corrected *p* value [1]**
P150/N250	Random intercept, random slope, quadratic slope, and age, volume and GAC score as predictors [$\chi 2$(7, N=116) = 334.78, *p*<0.0001]	Quadratic slope	*F* (1, 543.45) = 6.34	*p*=0.012*
N250/P350	Random intercept, random slope, quadratic slope, and volume and GAC score as predictors [$\chi 2$(5, N=116) = 355.49, *p*<0.0001]	Intercept	*F* (1, 287.38) = 5.25	*p*=0.046*
		Quadratic slope	*F* (1, 557.92) = 63.37	*p*<0.0004*
		ROI	*F* (3, 885.44) = 4.5	*p*=0.012*
		Group*Presentation* Volume	*F*(1, 372.05)=4.3	*p*=0.046*
P350/N450	Random intercept, random slope, quadratic slope, and age, volume and GAC score as predictors [$\chi 2$(7, N=116) = 423.02, *p*<0.0001]	Intercept	*F* (1, 248.89) = 28.47	*p*<0.0004*
		Quadratic slope	*F* (1, 527.62) = 329.06	*p*<0.0004*
		Age	*F* (1, 244.63) = 6.87	*p*=0.009*
		GAC score	*F* (1, 244.94) = 14.53	*p*<0.0004*

1. Holm-Bonferroni method. **p*<.05

Supplementary Table S10. Estimates of fixed effects by peak-to-peak measure (µV). LMM (2 groups x 4 presentations x 4 ROI). Coefficients for P150/N250 and P350/N450 are interpreted inversely because of the negative sign.

**Peak-to-peak measure**	**Effet**	***b* (SE*b*)**	**corrected *p* value***
P150/N250	Quadratic slope	-0.140(0.05)	*p*=0.024*
	Presentation*age	-0.62(0.038)	*p*=0.038*
N250/P350	Intercept	6.2(2.6)	*p*=0.020*
	Quadratic slope	0.53(0.07)	*p*<0.0004*
	ROI= left frontal	1.16(0.35)	*p*=0.003*
	Group=Macrocephalic*Presentation*GAC	0.18(.09)	*p*=0.043*
P350/N450	Intercept	-22.43(4.24)	*p*<0.0005*
	Presentation	15.58(6.5)	*p*=0.018*
	Quadratic slope	-1.67(0.09))	*p*<0.0005*

1. Holm-Bonferroni method. *p<.05

Note: (SE) Standard error.

Supplementary Table S11. Descriptive statistics (mean and standard deviation) for latency (ms) by group, component and ROI per stimulus of the standard sequence /a//a//a//i/.

		Group	/a/	/a/	/a/	/i/
P150	**Central**	Macrocephalic	133.67(37.91)	150.17(46.12)	153.85(49.97)	116.16(41.80)
		Normocephalic	128.98(48.46)	147.46(43.50)	130.62(49.92)	123.40(54.21)
	**Frontocentral**	Macrocephalic	147.27(36.88)	159.53(47.01)	147.94(44.13)	124.96(43.47)
		Normocephalic	143.12(45.76)	144.11(46.36)	138.58(52.20)	131.57(44.03)
	**Left frontal**	Macrocephalic	140.66(36.92)	154.18(40.90)	148.21(38.17)	132.32(41.58)
		Normocephalic	148.70(40.95)	156.15(49.72)	158.86(49.97)	144.03(50.65)
	**Right frontal**	Macrocephalic	146.28(37.30)	156.19(35.81)	145.79(43.69)	126.53(40.98)
		Normocephalic	146.68(41.69)	150.76(46.82)	154.84(49.74)	135.98(43.05)

		Group	/a/	/a/	/a/	/i/
N250	**Central**	Macrocephalic	201.57(58.59)	191.83(52.44)	218.59(53.22)	199.84(49.05)
		Normocephalic	203.05(62.26)	215.19(51.53)	215.89(60.84)	198.29(54.85)
	**Frontocentral**	Macrocephalic	210.06(56.98)	212.21(56.66)	223.37(42.87)	197.23(34.19)
		Normocephalic	215.71(63.85)	208.26(52.98)	222.94(57.31)	197.60(45.66)
	**Left frontal**	Macrocephalic	190.64(51.58)	199.20(50.00)	223.43(50.13)	194.13(38.32)
		Normocephalic	206.57(56.12)	217.31(53.09)	233.47(45.13)	204.28(44.13)
	**Right frontal**	Macrocephalic	207.68(51.60)	207.78(48.82)	217.89(42.20)	193.71(30.05)
		Normocephalic	210.85(51.82)	220.22(50.66)	231.36(44.06)	200.71(37.17)

		Group	/a/	/a/	/a/	/i/
P350	**Central**	Macrocephalic	267.07(63.36)	304.95(50.70)	308.45(64.24)	306.91(58.66)
		Normocephalic	283.84(81.37)	323.16(67.16)	311.16(77.26)	300.95(65.87)
	**Frontocentral**	Macrocephalic	287.01(57.49)	314.99(58.65)	313.90(62.54)	308.98(51.99)
		Normocephalic	294.63(62.29)	317.19(61.75)	317.58(69.07)	310.66(54.30)
	**Left frontal**	Macrocephalic	278.08(52.14)	301.88(61.59)	314.09(40.02)	303.82(48.13)
		Normocephalic	295.03(61.73)	317.54(60.64)	322.79(58.41)	316.92(57.87)
	**Right frontal**	Macrocephalic	278.56(60.04)	303.39(70.57)	303.51(43.01)	303.95(50.74)
		Normocephalic	293.31(58.21)	322.63(58.45)	331.15(63.27)	312.04(52.23)

		Group	/a/	/a/	/a/	/i/
N450	**Central**	Macrocephalic	474.08(59.98)	422.38(70.97)	450.71(62.53)	458.45(66.90)
		Normocephalic	474.34(59.14)	438.19(79.58)	457.15(75.75)	452.90(68.71)
	**Frontocentral**	Macrocephalic	479.02(51.04)	438.27(65.30)	452.74(66.81)	469.09(66.20)
		Normocephalic	479.25(66.11)	446.18(80.21)	462.40(83.90)	458.42(69.53)
	**Left frontal**	Macrocephalic	459.25(61.76)	446.98(68.09)	448.49(50.12)	472.43(71.67)
		Normocephalic	478.23(65.93)	447.28(71.74)	463.47(70.62)	476.33(73.81)
	**Right frontal**	Macrocephalic	477.51(60.15)	448.60(60.52)	469.46(64.24)	476.15(74.27)
		Normocephalic	472.66(68.69)	450.04(71.25)	463.48(73.85)	465.09(72.19)

Supplementary Table S12. Best model fit statistics by component (latency, ms). LMM(2Groups x 4 presentations x 4 ROI).

Component	Best fit model	Effect	*F* (df)	corrected *p* value [1]
P150	Random intercept, random slope, quadratic slope, and age, volume and GAC score as predictors [$\chi 2$(5, N=116) = 822.04, p<0.0001]	Presentation<br>Quadratic slope	*F* (1, 542.16) = 13.8<br>*F* (1, 583.8) = 34.91	*p*<0.0002 *<br>*p*<0.0002*
N250	Random intercept, quadratic slope, and age, volume and GAC score as predictors [$\chi 2$(6, N=116) = 853.27, p<0.0001]	Quadratic slope	*F* (1, 647.28) = 24.57	*p*<0.0001*
P350	Random intercept, random slope, quadratic slope, and age, volume and GAC score as predictors [$\chi 2$(5, N=116) = 853.27, p<0.0001]	Intercept<br>Presentation<br>Quadratic slope	*F* (1, 457.76) = 4.99<br>*F* (1, 522.92) = 10.53<br>*F* (1, 577.31) = 24.05	*p*=0.026*<br>*p*=0.002*<br>*p*<0.0003*
N450	Random intercept, random slope, quadratic slope, and age, volume and GAC score as predictors [$\chi 2$(5, N=116) = 881.22, p<0.0001]	Intercept<br>Quadratic slope<br>Group*Presentation*GAC<br>Group*Presentation*Volume*GAC	*F* (1, 525.33) = 66.27<br>*F* (1, 586.58) = 24.88<br>*F* (1, 169.45) = 4.63<br>*F* (2, 169.42) = 4.13	*p*<0.0004*<br>*p*<0.0004*<br>*p*=0.036*<br>*p*=0.036*

1. Holm-Bonferroni method. **p*<.05

Note: (df) degree of freedom

Supplementary Table S13. Estimates of fixed effects by component (latency, ms). LMM (2 groups x 4 presentations x 4 ROIs).

Component	Effet	*b* (SE*b*)	corrected *p* value*
P150	Presentation	102.66(24.7)	$p<0.0003$*
	Quadratic slope	-5.30(1.3)	$p<0.0003$*
N450	Intercept	543.6(65.9)	$p<0.0007$*
	Presentation	- 218.7(80.1)	$p=0.035$*
	Quadratic slope	9.47(1.9)	$p<0.0007$*
	ROI=Frontocentral*Presentation* Age	4.55(1.97)	$p=0.044$*
	Group= Normocephalic*Presentation*Volume	0.263(.11)	$p=0.044$*
	Group=Normocephalic*Presentation*Volume*GAC	-.0026(.001)	$p=0.044$*

1. Holm-Bonferroni method. *$p<.05$

Note: (SE) Standard error.

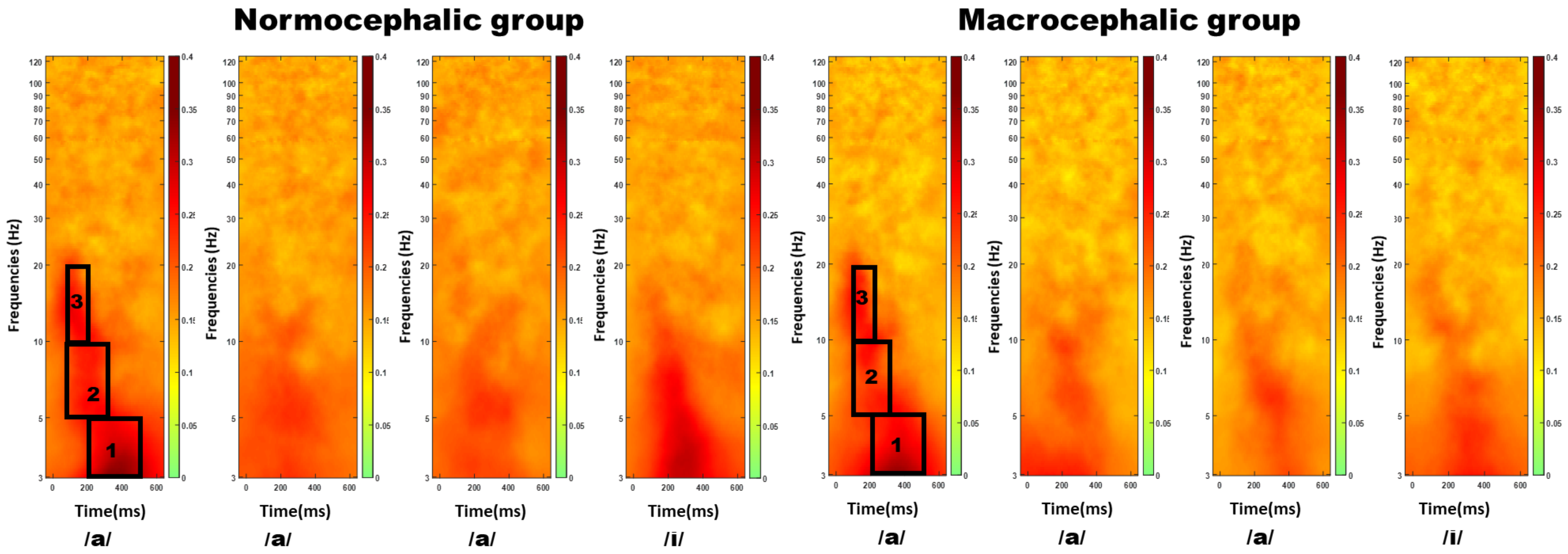

Supplementary Figure S2. Frontocentral region: Average inter-trial phase coherence across the standard sequence /a/a/a/i/ by group. The x-axis represents time, while the y-axis displays frequency. Black squares are showing the selected time-frequency windows: 1) 3-5Hz(200-500ms); 2) 5-10Hz (100-300ms and 3)10-20Hz(100-200ms).

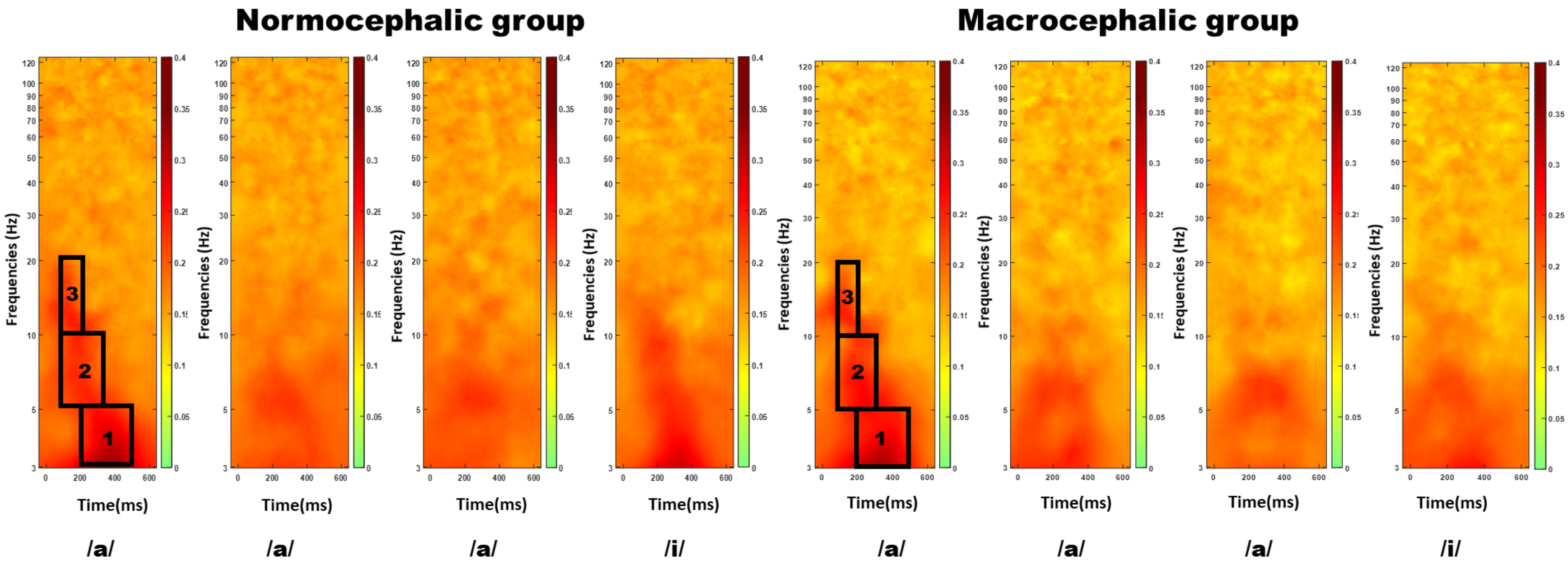

Supplementary Figure S3. Left frontal region: Average inter-trial phase coherence across the standard sequence /a/a/a/i/ by group. The x-axis represents time, while the y-axis displays frequency. Black squares are showing the selected time-frequency windows: 1) 3-5Hz(200-500ms); 2) 5-10Hz (100-300ms) and 3)10-20Hz(100-200ms).

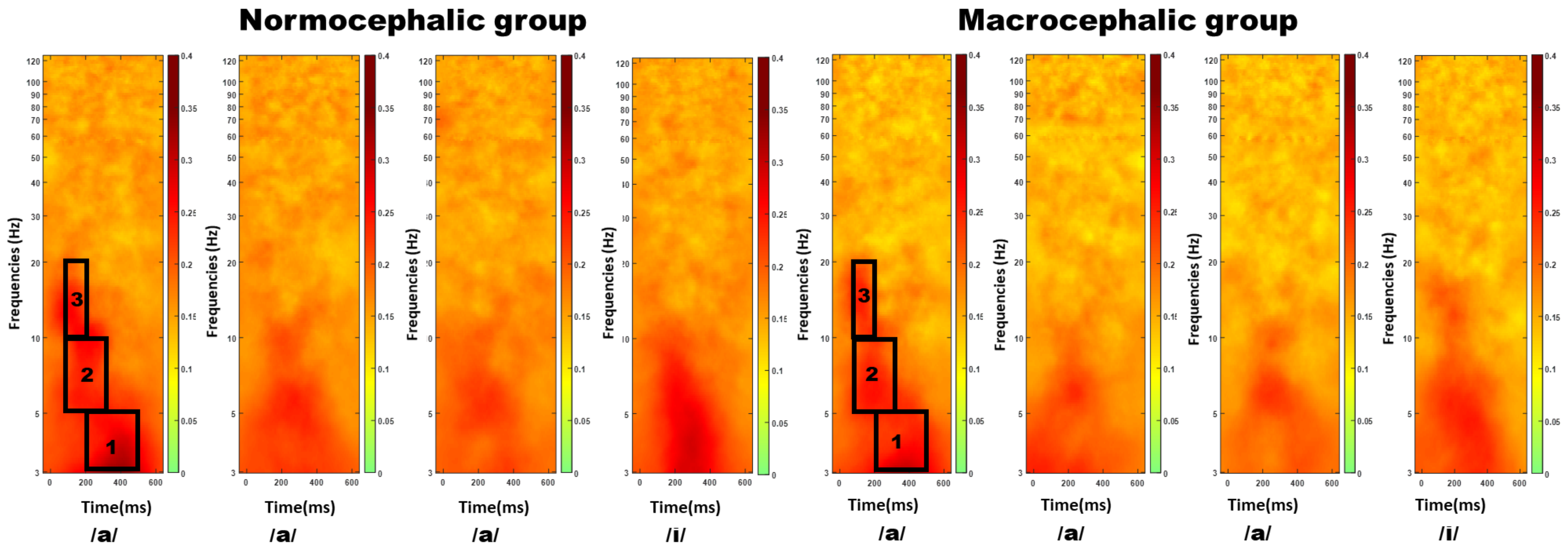

Supplementary Figure S4. Right frontal region: Average inter-trial phase coherence across the standard sequence /a/a/a/i/ by group. The x-axis represents time, while the y-axis displays frequency. Black squares are showing the selected time-frequency windows: 1) 3-5Hz(200-500ms); 2) 5-10Hz (100-300ms) and 3)10-20Hz(100-200ms).

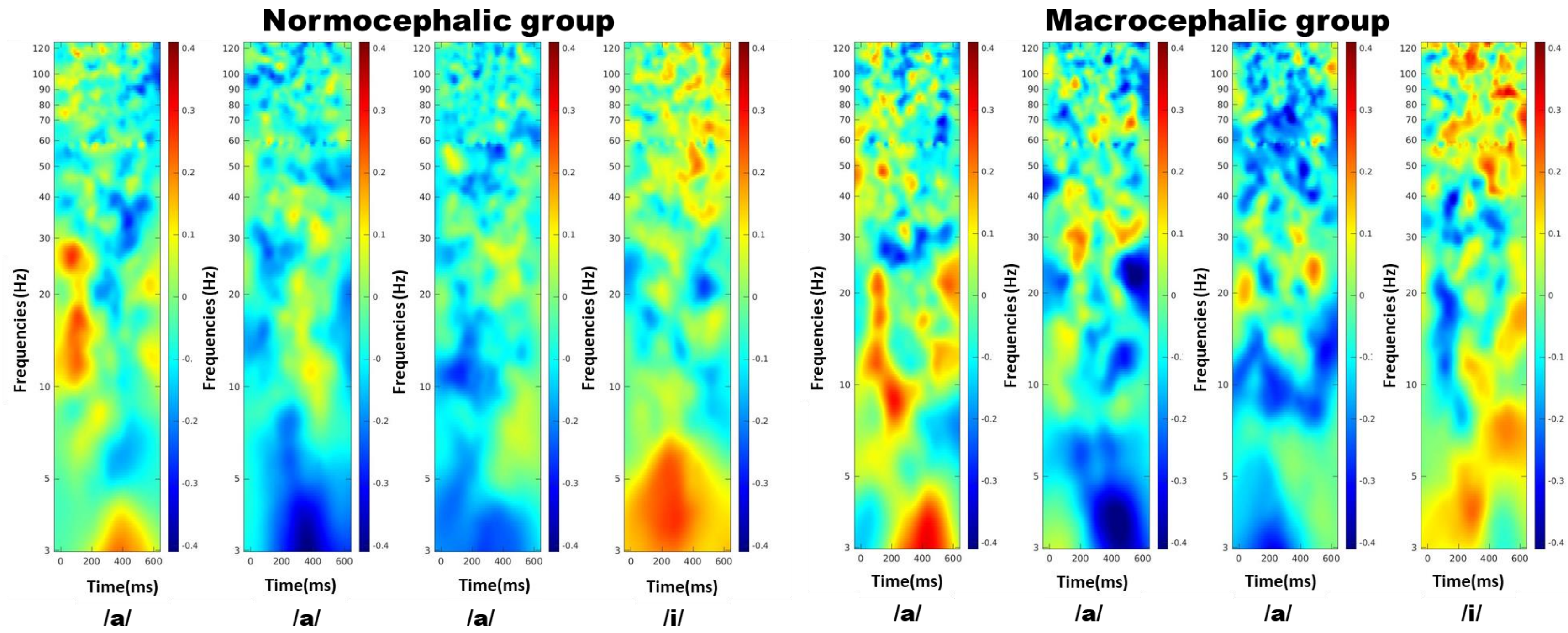

Supplementary Figure S5. Frontocentral region: changes in spectral power across the standard sequence by group. The x-axis represents time, while the y-axis displays frequency. The side bar shows a color representation in decibels (db).

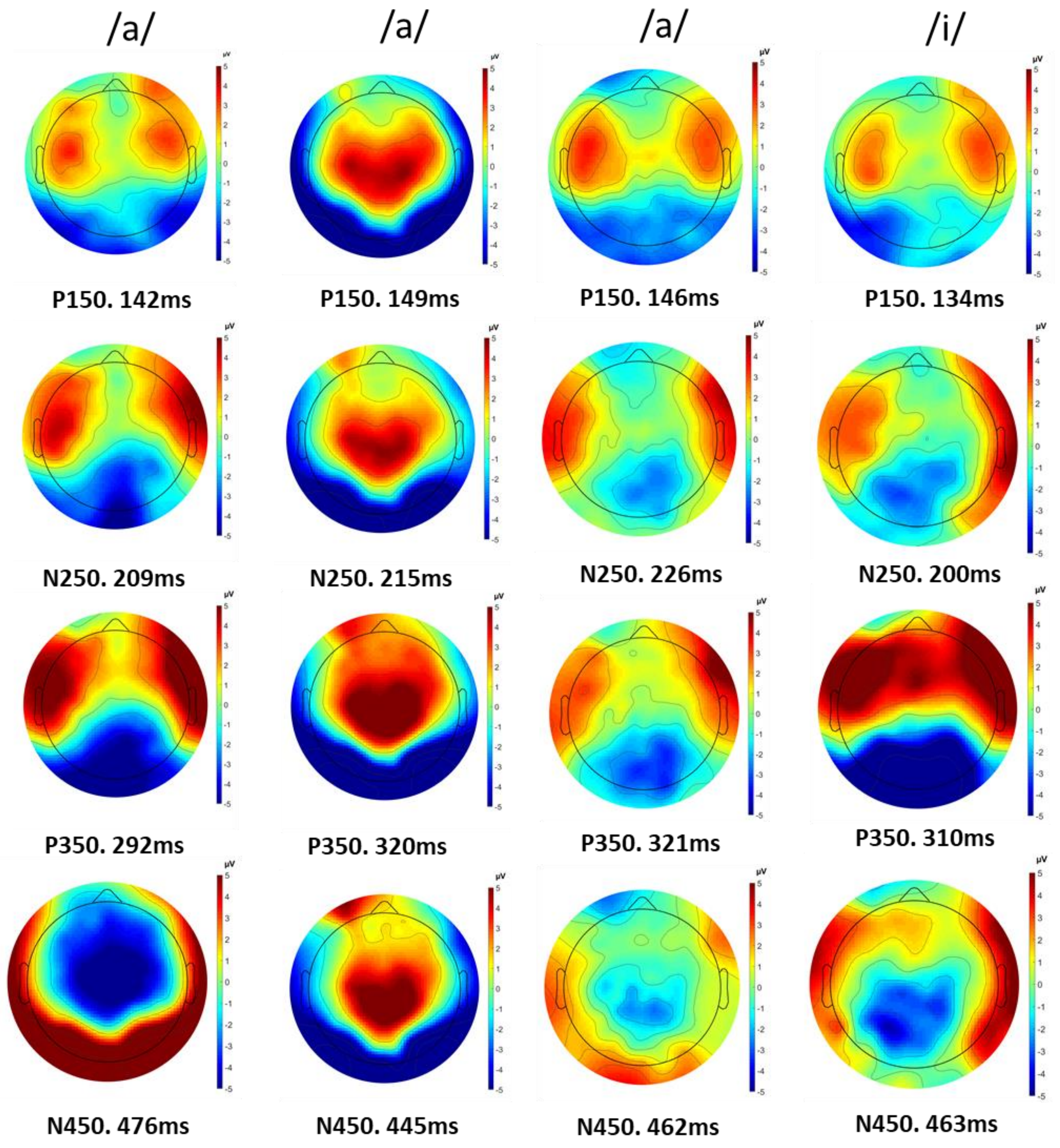

Supplementary Figure S6. Normocephalic group. Topographic maps by component (rows) for each stimulus of the standard sequence (columns). Each topographic map corresponds to the mean values at the average latency for P150, N250, P350 and N450 components per stimulus.

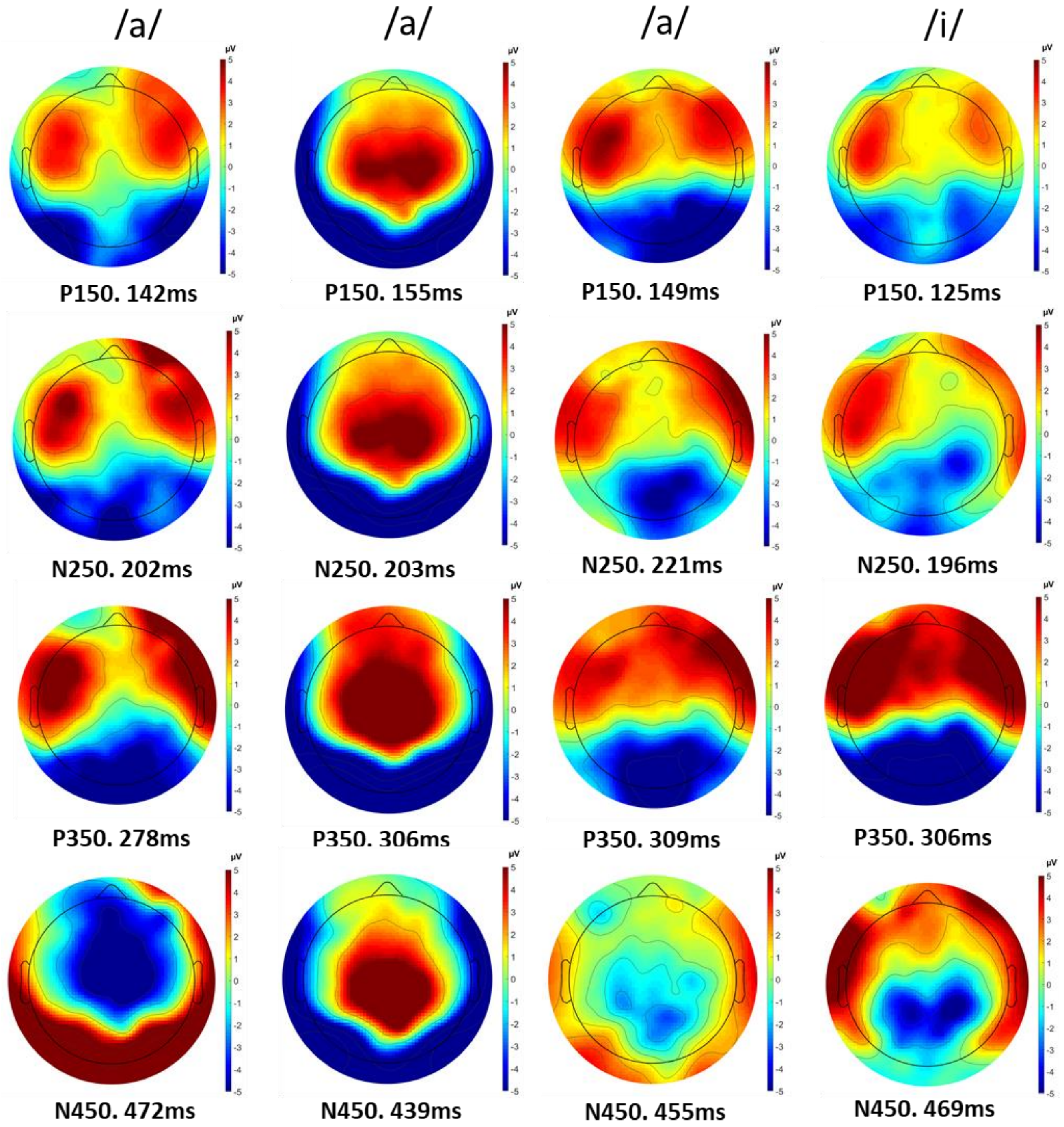

Supplementary Figure S7. Macrocephalic group. Topographic maps by component (rows) for each stimulus of the standard sequence (columns). Each topographic map corresponds to the mean values at the average latency for P150, N250, P350 and N450 components per stimulus.

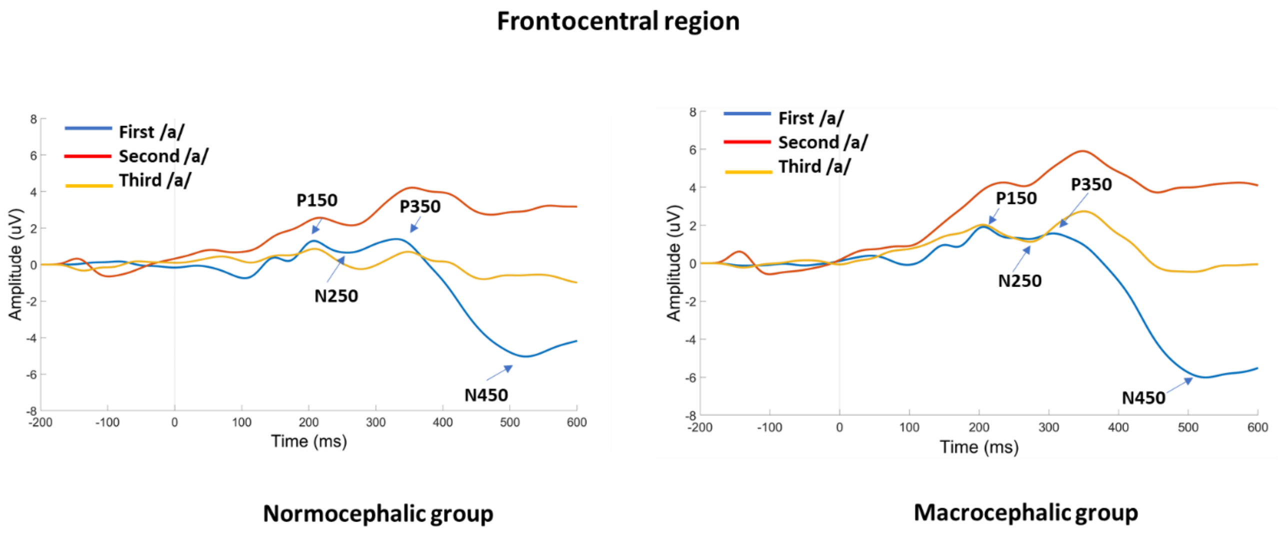

Supplementary Figure S8. Grand Average ERPs waveforms in the frontocentral region by group for the first, second and third /a/ presentation. A low-pass filter at 20Hz was used only for illustration. The x-axis represents time, while the y-axis displays amplitude (µV). The baseline is set to -200-0ms relative to stimulus onset for all waveforms. P150, N250, P350 and N450 peaks for the first /a/ presentation are indicated. In our total sample, a repetition suppression effect was only observed as a decrease in P350/N450 peak-to-peak amplitude and as a decrease in N450 latency. No difference between groups was found in these effects.

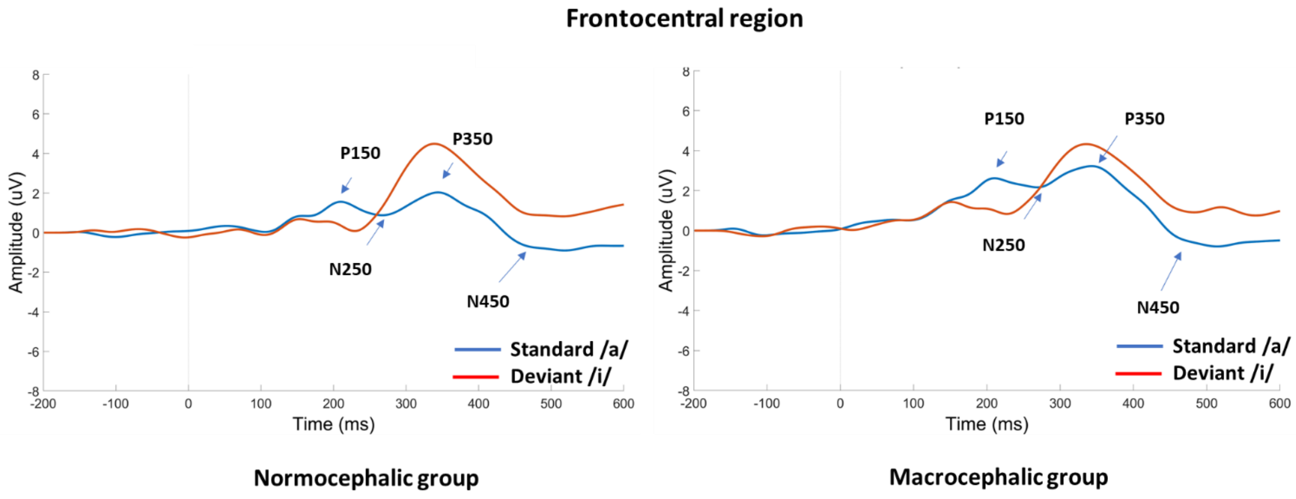

Supplementary Figure S9. Average of the three /a/ presentations (standard) vs local deviant /i/. P150, N250, P350 and N450 peaks for the standard waveform are indicated. A low-pass filter at 20Hz was used only for illustration. In our total sample a significant change detection response was observed in terms of P150/N250, N250/P350 and P350/N450 peak-to-peak amplitudes.

$$M(t,f) = W * S = \int_t W(t - a/b, f)\, S(t).\, dt$$

Supplementary Equation S1. Specific wavelet convolution expression. This convolution provided TF power maps for each stimulus repetition, S is the EEG signal as a function of time, W is the complex Morlet's wavelet, and a and b are the translation and dilatation parameters. Lastly, *M(t, f)* is a matrix of complex values given for time (t) and frequency (f) (Tallon-Baudry and Bertrand, 1999).

$$P(t,f) = 10 \log_{10}(|M(t,f)|^2)$$

Supplementary Equation S2. ERSP were computed for each trial, and then averaged across trials, using the amplitude and phase given by Morlet's wavelet transformation where *|M(t, f)|2* is the squared absolute value for every complex vector of a TF map, the logarithmic transformation converts values in decibels and thus P(t,f) denotes TF power in terms of decibels.
